# Child Mortality in Rural Malawi: HIV Closes the Survival Gap between the Socio-Economic Strata

**DOI:** 10.1371/journal.pone.0011320

**Published:** 2010-06-28

**Authors:** Andreas Jahn, Sian Floyd, Nuala McGrath, Amelia C. Crampin, Lackson Kachiwanda, Venance Mwinuka, Basia Zaba, Paul E. M. Fine, Judith R. Glynn

**Affiliations:** 1 Department of Epidemiology and Population Health, London School of Hygiene & Tropical Medicine, London, United Kingdom; 2 Africa Centre for Health and Population Studies, University of KwaZulu Natal, Mtubatuba, South Africa; 3 Karonga Prevention Study, Chilumba, Karonga, Malawi; Stanford University, United States of America

## Abstract

**Background:**

As HIV-related deaths increase in a population the usual association between low socioeconomic status and child mortality may change, particularly as death rates from other causes decline.

**Methods/Principal Findings:**

As part of a demographic surveillance system in northern Malawi in 2002-6, covering a population of 32,000, information was collected on socio-economic status of the households. Deaths were classified as HIV/AIDS-related or not by verbal autopsy. Poisson regression models were used to assess the association of socio-economic indicators with all-cause mortality, AIDS-mortality and non-AIDS mortality among children. There were 195 deaths in infants, 109 in children aged 1–4 years, and 38 in children aged 5–15. All-cause child mortality in infants and 1–4 year olds was similar in households with higher and lower socio-economic status. In infants 13% of deaths were attributed to AIDS, and there were no clear trends with socio-economic status for AIDS or non-AIDS causes. For 1–4 year olds 27% of deaths were attributed to AIDS. AIDS mortality was higher among those with better built houses, and lowest in those with income from farming and fishing, whereas non-AIDS mortality was higher in those with worse built houses, lowest in those with income from employment, and decreased with increasing household assets.

**Conclusions/Significance:**

In this population, since HIV infection among adults was initially more common among the less poor, childhood mortality patterns have changed. The usual gap in survival between the poor and the less poor has been lost, but because the less poor have been disproportionately affected by HIV, rather than because of relative improvement in the survival of the poorest.

## Introduction

Associations between socioeconomic status and mortality have long been recognised. Among rich and poor populations even fine gradations in relative poverty have been associated with differences in survival.[1], [2] It is less clear if this has always been the case.[3] Pre-1900, being able to reach or afford health care would have been of limited benefit, and in predominantly rural communities, exposure to crowds and infectious diseases may have been limited among the poor.

The HIV epidemic has produced some parallels with this historical situation. In many populations HIV initially affected the better off, more educated and more mobile sectors of the community.[4], [5], [6] And before the widespread availability of effective antiretrovirals and prevention of mother-to-child transmission, access to health care had limited impact on HIV-related mortality.

In populations with high HIV prevalence, HIV is the predominant cause of death in young adults, and an important cause in young children–directly, through vertical transmission, and indirectly, through orphanhood and parental illness.[7] This may be sufficient to attenuate, or even reverse, the expected associations between socio-economic status and mortality.

In a rural area of northern Malawi we have documented mortality patterns through a demographic surveillance system over the period leading up to the wide-spread use of antiretrovirals. HIV infection was first recorded in this area in 1982.[8] The prevalence increased slowly in the 1980s, and reached a plateau of 10–11% in adults from the mid 1990s.[8], [9]

In the adult population in this area, in the early stages of the HIV epidemic in 1988–90, HIV infection was more common among non-farmers, those with higher education and among people born outside the district.[9] A decade later, in a stable generalised epidemic, occupation other than farming remained associated with higher prevalence, and HIV infection was more common among adults with higher and with no education, compared with individuals with only primary education.[9]

We have already shown that just before the introduction of antiretrovirals, two thirds of deaths in those aged 15–59 were attributable to AIDS.[10] We now assess the association between socio-economic factors and child mortality in this population.

## Methods

Ethics statement: The work was approved by the Health Sciences Research Committee, Malawi and by the ethics committee of the London School of Hygiene and Tropical Medicine, UK. The methods of the demographic surveillance system have been described in detail elsewhere.[11] Before the start of the study the Traditional Authority that covers the area, and all village headmen and traditional advisors in the study area were informed about the aims of the study and the nature of the data to be collected, and their approval and verbal consent was sought. All household members were given a similar explanation and interviews were only conducted if verbal consent was given by the household head and by the respective household members. The consent was recorded by the interview sheet being filled. Refusals were recorded in field registers. During the baseline census 15 households did not provide verbal consent and have consequently been excluded from the study. The data for this study come from the basic demographic surveillance for which the ethics committees agreed that written consent was not needed.

After an initial house-to-house census, a continuous registration system was set up in a population of around 32,000. Trained community members (“village informants”) recorded births, deaths and migrations against pre-printed lists for their area. Each informant covered 15–60 households. Births and deaths were followed up monthly, and migrations annually, by project staff. A re-census after two years showed that the routine system had registered 97% of births, 99% of deaths, and 92% of migrations.[11]

Verbal autopsy interviews were conducted in the local language by a medical assistant or health worker with the mother or most immediate caregiver. Verbal informed consent was obtained before the interview. The verbal autopsies were similar to the INDEPTH tool,[12] an adaptation of the standard WHO questionnaire, with different forms for neonatal and other child deaths. Information from patient-held health documents and hospital records was used when available. Three individuals (doctors or clinical officers) coded the results independently. Information on maternal HIV status from previous or other concurrent studies in the population was used by the reviewers where available. HIV/AIDS was assigned as a cause of death if clinical symptoms suggested immunosuppression in the absence of other obvious causes (e.g. underlying malnutrition), taking account of any available information on maternal HIV infection or AIDS death, and any prior diagnoses of suspected HIV infection. Discrepantly coded cases were discussed, and resolved where possible. Socio-economic and other household data were not available to the reviewers of the verbal autopsies.

Socio-economic data were collected at the household level during the baseline census and at the registration visit for in-migrating, newly-formed, or moved households. Several measures of socio-economic status were used: education level of the mother and father; main source of income of the household; household possessions; and dwelling construction. Parental education status was only known if the parent was included in the baseline census or registration visit, and so was missing for parents who had died before this time, and for those who were not normally resident in the demographic surveillance area.

Previous field work and a pilot census had shown that there was little formal employment and that for their livelihood households commonly relied on a variety of different income-generating activities in addition to subsistence agriculture. Specific income-generating activities were initially collected as free-text descriptions during the pilot census and then classified into 11 categories: sale of own agricultural produce, sale of own fish, regular employment, small scale trading, piecework, manufacturing, preparation and sale of snacks or drinks (commonly doughnuts, beer or local spirit), letting of houses or land, providing a service (waiter, hairdresser, etc.), gathering natural products (firewood, charcoal, grass for thatching, etc.). Households were asked to name all income generating activities they had engaged in over the last 12 months and then to rank the listed activities by importance. For the analysis, the main source of household income was grouped into 6 categories (farming, fishing, employment, piecework, small trading and other).

A standard inventory of household possessions appropriate to the setting (bicycle, oxcart, motorbike, watch or clock, radio, mosquito net, canoe, fishing net, cattle) was converted into an approximate monetary value. To avoid over-estimating the wealth of households with many cattle, the asset category was calculated first without the cattle, and then increased by a maximum of one or two categories according to the number of cattle owned.

Dwelling construction was classified according to the materials used to make the roof, walls and floor. The “best” houses had concrete floors, iron sheet or tile roofs, and burnt brick or concrete walls. The “worst” had mud floors, no iron sheet or tiles on the roof, and walls of unburnt brick, mud, or other materials.

### Statistical analysis

All analyses were carried out in Stata 10.0 (Stata Corporation, USA). Observation time for each child was defined from the time they were first seen in the baseline census, from birth (if the mother was a member of a household in the observation area at the time of birth) or from the time of migration into the area after the baseline census. Observation time ended at the time of death (if the child was still a member in a household in the area at the time of death) or at the time of migration out of the surveillance area. Multiple episodes of observation and gaps were allowed if children moved out of the surveillance area and later returned.

For the estimation of AIDS mortality, the failure event was death from AIDS, while deaths from all other causes (known or unknown) were censored. For the estimation of mortality from other causes (‘non-AIDS mortality’), the failure event was death from causes other than AIDS, and deaths from AIDS and from unknown causes were censored.

The age groups <1, 1–4 and 5–14 years were analysed separately as mortality rates are dramatically different in these age groups. The reliability of verbal autopsy and the relative contribution of HIV to mortality are also likely to vary.

Poisson regression models were used to assess the association of mortality with background characteristics. Age, sex and area of residence were considered as *a priori* confounders. The overall strength of association for each variable was assessed using the likelihood ratio test, comparing the model with and without the variable of interest. Formal tests for trend across categories were conducted where appropriate. Analyses were repeated using Cox models, to allow for changing rates by age, and using robust standard errors to allow for non-independence of data from children with the same mother. The results were almost identical; only the Poisson regression results are presented.

## Results

Between August 2002 and the end of February 2006, a total of 20,389 children under 15 years were observed in the study population, of whom 10,415 were under 5 years and 5,237 were under 1 year. There were 195 deaths in infants (<1 year), 109 in those aged 1–4 years, and 38 in those aged 5–14 years.

Tables 1 and 2 show the association of socio-economic factors with all cause mortality rates by age group. There were small numbers of children with missing information on socio-economic status, but these children tended to have higher mortality rates. There was little evidence of the usual trend of higher mortality in lower socioeconomic groups in any age group. Because of the low numbers of deaths in the older children, associations with socio-economic status were only explored further in those under 5 years.

**Table 1:** Age-specific mortality (per 1000 person years observation [PYO]) from all causes by parental education.

	0–11 months			1–4 years			5–14 years		
	D/PYO	Rate	95% CI	D/PYO	Rate	95% CI	D/PYO	Rate	95% CI
Mother's education									
None/primary 1–4	20/348	**57.4**	37.1–89.0	13/1288	**10.1**	5.86–17.4	2/2903	**0.69**	0.17–2.76
primary 5–8	131/2393	**54.8**	46.1–65.0	64/7964	**8.04**	6.29–10.27	20/13,302	**1.50**	0.97–2.33
secondary +	38/765	**49.6**	36.1–68.2	22/2131	**10.3**	6.80–15.7	3/1937	**1.55**	0.50–4.80
unknown	6/57	**110**	47.5–235	10/620	**16.1**	8.68–30.0	13/4910	**2.65**	1.54–4.56
Father's education									
None/primary 1–4	8/203	**39.4**	19.7–78.9	11/659	**18.8**	9.24–30.1	0/1400	**0.0**	
primary 5–8	104/1700	**61.2**	50.5–74.1	43/5266	**8.16**	6.06–11.0	11/9068	**1.21**	0.67–2.19
secondary +	51/1295	**39.4**	29.9–51.8	37/3732	**9.91**	7.18–13.7	9/4862	**1.85**	0.96–3.56
unknown	32/366	**87.5**	61.9–124	18/2345	**7.68**	4.84–12.2	18/7724	**2.33**	1.47–3.70

**Table 2:** Age-specific mortality (per 1000 person years observation [PYO]) from all causes by household characteristics.

	0–11 months			1–4 years			5–14 years		
	D/PYO	Rate	95% CI	D/PYO	Rate	95% CI	D/PYO	Rate	95% CI
Household assets									
$ 0-	43/686	**62.7**	46.5–84.5	27/2155	**12.5**	8.59–18.3	6/4007	**1.50**	0.67–3.33
$5-	44/772	**57.0**	42.4–76.6	21/2463	**8.53**	5.56–13.1	10/4214	**2.37**	1.28–4.41
$10-	57/1226	**46.5**	35.9–60.3	41/4214	**9.73**	7.16–13.2	13/7677	**1.69**	0.98–2.92
$50-	24/549	**43.7**	29.3–65.3	12/1966	**6.10**	3.47–10.7	4/4152	**0.96**	0.36–2.57
$ 150-	22/268	**82.1**	54.1–124	5/1020	**4.90**	2.04–11.8	5/2583	**1.94**	0.81–4.65
unknown	5/62	**80.7**	33.5–194	3/184	**16.3**	5.26–50.6	0/417		
Dwelling class									
1 (Best)	27/568	**47.5**	32.6–69.3	19/2079	**9.14**	5.83–14.3	10/4852	**2.06**	1.11–3.83
2	25/407	**61.9**	41.9–91.7	12/1412	**8.50**	4.83–15.0	9/3091	**2.91**	1.51–5.60
3	58/1119	**51.8**	40.1–67.0	33/3862	**8.54**	6.07–12.0	8/7416	**1.08**	0.54–2.16
4 (Worst)	79/1416	**55.8**	44.8–69.6	44/4450	**9.89**	7.36–13.3	11/7270	**1.51**	0.84–2.73
unknown	6/56	**106.8**	48.0–238	1/200	**5.01**	0.71–35.6	0/422	**0.0**	-
Household main source of income									
farming	78/1482	**52.6**	42.2–65.7	48/5012	**9.58**	7.22–12.7	10/9479	**1.06**	0.57–1.96
employment	19/483	**39.3**	25.1–61.7	11/1785	**6.16**	3.41–11.1	6/3882	**1.55**	0.69–3.44
piecework	17/357	**47.6**	29.6–76.5	9/1089	**8.27**	4.30–15.9	5/1583	**3.16**	1.31–7.59
small trading	24/452	**53.1**	35.6–79.2	12/1412	**8.50**	4.83–15.0	5/2474	**2.02**	0.84–4.85
fishing	23/329	**69.9**	46.5–105.3	7/1072	**6.53**	3.11–13.7	4/1850	**2.16**	0.81–5.76
other	32/416	**76.9**	54.4–109	21/1494	**14.1**	9.16–21.6	7/3475	**2.01**	0.96–4.23
unknown	2/44	**46.0**	11.5–184	1/138	**7.26**	1.02–51.5	1/309	**3.24**	0.46–23.0

**Figure 1:**
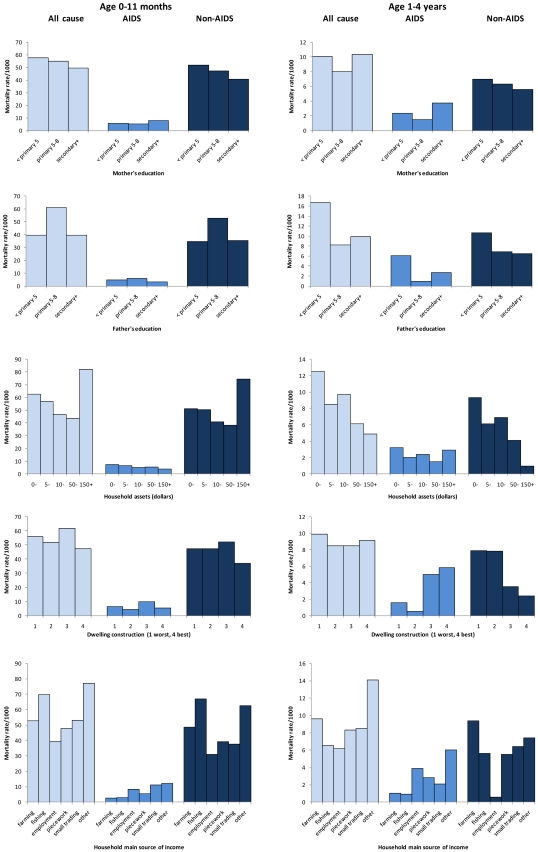
Mortality rates by socio-economic status and cause in infants and children age 1–4 years, Karonga District Malawi, 2002-6.

In Tables 3, 4, 5 and 6 and figure 1, deaths are shown overall and separately for those attributed by reviewers to AIDS and to non-AIDS. Associations with socio-economic factors are shown separately for infants (tables 3 and 4) adjusted for sex and area of residence, and for children aged 1–4 years (tables 5 and 6), adjusted for year of age, sex and area of residence.

**Table 3:** Association of all cause, AIDS and non-AIDS mortality with parental education in infants.

	All cause mortality				AIDS mortality				Non-AIDS mortality			
	D/PYO	Rate	Rate Ratio	[95% CI]	D/PYO	Rate	Rate Ratio	[95% CI]	D/PYO	Rate	Rate Ratio	[95% CI]
Mother's education												
None/primary 1–4	20/348	57.4	**1.14**	0.66–1.98	2/348	5.7	**0.91**	0.18–4.60	18/348	51.7	**1.24**	0.69–2.24
primary 5–8	131/2393	54.8	**1.10**	0.77–1.59	13/2393	5.4	**0.79**	0.30–2.10	113/2393	47.2	**1.15**	0.77–1.72
secondary +	38/765	49.6	**1**	*P = 0.8*	6/765	7.8	**1**	*P = 0.9*	31/765	40.5	**1**	*P = 0.7 [0.4 tr]*
Father's education												
None/primary 1–4	8/203	39.4	**0.95**	0.45–2.01	1/203	4.9	**1.99**	0.22–18.0	7/203	34.5	**0.91**	0.41–2.02
primary 5–8	104/1700	61.2	**1.55**	1.10–2.17	10/1700	5.9	**2.11**	0.66–6.79	90/1700	52.9	**1.48**	1.03–2.11
secondary +	51/1295	39.4	**1**	*P = 0.02*	4/1295	3.1	**1**	P = 0.4	46/1295	35.5	**1**	*P = 0.06*

Rate ratios are adjusted for sex and area. Deaths with unknown causes are censored in the analysis of AIDS and non-AIDS mortality.

**Table 4:** Association of all cause, AIDS and non-AIDS mortality with household characteristics in infants.

	All cause mortality				AIDS mortality				Non-AIDS mortality			
	D/PYO	Rate	Rate Ratio	[95% CI]	D/PYO	Rate	Rate Ratio	[95% CI]	D/PYO	Rate	Rate Ratio	[95% CI]
Household assets												
$ 0-	43/686	62.7	**1**	*P = 0.2*	5/686	7.3	**1**	*P = 1.0*	35/686	51.0	**1**	*P = 0.2*
$5-	44/772	57.0	**0.91**	0.60–1.39	5/772	6.5	**0.86**	0.25–2.98	39/772	50.5	**1.00**	0.63–1.58
$10-	57/1226	46.5	**0.75**	0.51–1.12	6/1226	4.9	**0.70**	0.21–2.31	50/1226	40.8	**0.81**	0.53–1.25
$50-	24/549	43.7	**0.70**	0.43–1.16	3/549	5.5	**0.73**	0.17–3.09	21/549	38.3	**0.76**	0.44–1.31
$ 150-	22/268	82.1	**1.31**	0.78–2.20	1/268	3.7	**0.59**	0.069–5.08	20/268	74.6	**1.45**	0.83–2.52
Dwelling class												
1 (Best)	27/568	47.5	**0.84**	0.53–1.31	3/568	5.3	**0.68**	0.18–2.58	21/568	36.9	**0.78**	0.47–1.29
2	25/404	61.9	**1.10**	0.69–1.74	4/404	9.9	**1.38**	0.42–4.59	21/404	52.0	**1.10**	0.67–1.81
3	58/1119	51.8	**0.89**	0.63–1.26	5/1119	4.5	**0.61**	0.20–1.85	53/1119	47.4	**0.97**	0.67–1.40
4 (Worst)	79/1416	55.8	**1**	*P = 0.7*	9/1416	6.4	**1**	*P = 0.6*	67/1416	47.3	**1**	*P = 0.7*
Household main source of income												
farming	78/1482	52.6	**1**	P = 0.1	4/1482	2.7	**1**	*P = 0.2*	78/1482	48.6	**1**	*P = 0.1*
employment	19/483	39.3	**0.83**	0.50–1.40	4/483	8.3	**3.35**	0.78–14.4	19/483	31.1	**0.70**	0.40–1.24
piecework	17/357	47.6	**0.98**	0.58–1.67	2/357	5.6	**2.25**	0.40–12.6	17/357	39.2	**0.86**	0.48–1.54
small trading	24/452	53.1	**1.13**	0.70–1.82	5/452	11.1	**4.57**	1.15–18.2	24/452	37.6	**0.85**	0.49–1.47
fishing	23/329	69.9	**1.51**	0.92–2.49	1/329	3.0	**1.22**	0.13–11.7	23/329	66.9	**1.56**	0.93–2.61
other	32/416	76.9	**1.65**	1.07–2.54	5/416	12.0	**4.83**	1.22–19.2	32/416	62.5	**1.41**	0.88–2.26

Rate ratios are adjusted for sex and area. Deaths with unknown causes are censored in the analysis of AIDS and non-AIDS mortality.

**Table 5:** Association of all cause, AIDS and non-AIDS mortality with parental education in children 1–4 years of age.

	All cause mortality				AIDS mortality				Non-AIDS mortality			
	D/PYO	Rate	Rate Ratio	[95% CI]	D/PYO	Rate	Rate Ratio	[95% CI]	D/PYO	Rate	Rate Ratio	[95% CI]
Mother's education												
None/primary 1–4	13/1288	10.1	**0.99**	0.49–1.99	3/1288	2.33	**0.88**	0.23–3.41	9/1288	7.00	**1.13**	0.47–2.71
primary 5–8	64/7964	8.0	**0.80**	0.49–1.31	12/7964	1.51	**0.50**	0.20–1.24	50/7964	6.28	**1.09**	0.58–2.06
secondary +	22/2131	10.3	**1**	P = 0.06	8/2131	3.75	**1**	P = 0.3	12/2131	5.63	**1**	*p = 1.0*
Father's education												
None/primary 1–4	11/659	16.7	**1.69**	0.86–3.33	4/659	6.1	**3.02**	0.94–9.76	7/659	10.6	**1.52**	0.65–3.55
primary 5–8	43/5266	8.2	**0.82**	0.53–1.28	5/5266	0.95	**0.43**	*0.15–1.28*	36/5266	6.84	**1.00**	*0.59–1.69*
secondary +	37/3732	9.9	**1**	P = 0.1	10/3732	2.7	**1**	*P = 0.02*	24/3732	6.43	**1**	*P = 0.6*

Rate ratios are adjusted for sex, age in one-year intervals, and area. Deaths with unknown causes are censored in the analysis of AIDS and non-AIDS mortality.

**Table 6:** Association of all cause, AIDS and non-AIDS mortality with household characteristics in children 1–4 years of age.

	All cause mortality				AIDS mortality				Non-AIDS mortality			
	D/PYO	Rate	Rate Ratio	[95% CI]	D/PYO	Rate	Rate Ratio	[95% CI]	D/PYO	Rate	Rate Ratio	[95% CI]
Household assets												
$ 0-	27/2155	12.5	**1**	P = 0.2 [0.03tr]	7/2155	3.2	**1**	*p = 0.8*	20/2155	9.28	**1**	*P = 0.02 [0.006tr]*
$5-	21/2463	8.5	**0.66**	0.37–1.28	5/2463	2.0	**0.59**	0.19–1.86	15/2463	6.09	**0.65**	0.33–1.26
$10-	41/4214	9.7	**0.79**	0.48–1.28	10/4214	2.4	**0.75**	0.28–1.97	29/4214	6.88	**0.76**	0.43–1.34
$50-	12/1966	6.1	**0.52**	0.26–1.03	3/1966	1.5	**0.46**	0.12–1.79	8/1966	4.07	**0.48**	0.21–1.10
$ 150-	5/1020	4.9	**0.40**	0.15–1.05	3/1020	2.9	**1.02**	0.26–3.95	1/1020	0.98	**0.11**	0.015–0.81
Dwelling class												
1 (Best)	19/2079	9.1	**1.03**	0.59–1.80	12/2079	5.8	**3.63**	1.35–9.77	5/2079	2.4	**0.35**	0.13–0.90
2	12/1412	8.5	**0.97**	0.51–1.86	7/1412	5.0	**3.16**	1.07–9.34	5/1412	3.5	**0.53**	0.20–1.36
3	33/3862	8.5	**0.95**	0.60–1.50	2/3862	0.52	**0.34**	0.70–1.66	30/3862	7.8	**1.10**	0.20–1.36
4 (Worst)	44/4450	9.9	**1**	P = 1.0	7/4450	1.6	**1**	*P = 0.0004*	35/4450	7.9	**1**	*p = 0.03 [0.01 tr]*
Household main source of income												
farming	48/5012	9.6	**1**	P = 0.3	5/5012	1.0	**1**	*P = 0.02*	42/5012	8.4	**1**	*p = 0.005*
employment	11/1785	6.2	**0.69**	0.35–1.36	7/1785	3.9	**3.55**	1.07–11.7	1/1785	0.56	**0.074**	0.010–0.54
piecework	9/1089	8.3	**0.87**	0.42–1.78	3/1089	2.8	**2.66**	0.62–11.4	6/1089	5.5	**0.67**	0.28–1.59
small trading	12/1412	8.5	**0.93**	0.48–1.79	3/1412	2.1	**1.97**	0.45–8.59	9/1412	6.4	**0.81**	0.38–1.73
fishing	7/1072	6.5	**0.84**	0.36–1.92	1/1072	0.9	**0.78**	0.087–7.05	6/1072	5.6	**0.98**	0.39–2.43
other	21/1494	14.1	**1.57**	0.91–2.70	9/1494	6.0	**5.98**	1.90–18.9	11/1494	7.4	**0.94**	0.46–1.90

Rate ratios are adjusted for sex, age in one-year intervals, and area. Deaths with unknown causes are censored in the analysis of AIDS and non-AIDS mortality.

Among the infants, 22 deaths were attributed to AIDS, 167 to non-AIDS and 6 to unknown causes. The mother was known to be HIV positive by the time of birth in 7/22 AIDS deaths and 13/167 non-AIDS deaths. Mortality rates were higher in those whose fathers had 5–8 years primary education than in those with more or less (*p* = 0.02), and this pattern persisted in those thought to have died from non-AIDS causes (*p* = 0.06). There were no strong or statistically significant associations with any of the other measures of socio-economic status.

Among children aged 1–4 years, 27% of deaths were attributed to AIDS (28/104 with known causes; 5 unknown). Of the 28 AIDS deaths, 9 had a mother who was known to be HIV positive at the time of death, though only 4 were known to have been positive at the time of birth. Five of 76 children dying of non-AIDS causes had HIV positive mothers.

There was a “U-shaped” pattern of all-cause mortality associated with parental education, with the lowest levels in those with mothers or fathers with 5–8 years primary education (figure 1). When disaggregated by cause of death, the higher rates in those with more educated parents were only seen for AIDS mortality, but the associations were weak. A trend to lower mortality rates in those with more valuable household possessions was stronger for non-AIDS mortality (p trend = 0.006) than for all-cause mortality (p trend = 0.03); there was no evidence of a similar trend for AIDS mortality. While overall mortality was similar in those with different dwelling constructions (*p* = 1.0), mortality rates from AIDS were higher among those from the better built houses (*p* = 0.0004), and mortality rates from other causes were higher among those living in houses with inferior construction (*p* = 0.03). Overall mortality was similar in households with different main sources of income, but AIDS mortality was lowest in those with income from farming and fishing, and highest in those with income from employment and “other” sources, and non-AIDS mortality was lowest in those with income from employment. “Other income sources” included activities such as providing services (hairdressing, waitressing, etc.), producing and selling snacks or beer and relying on support from outside relatives. Among the 1–4 year olds who died, 18% of AIDS deaths and 75% of non-AIDS deaths were in those with the main family income from farming.

## Discussion

We have shown a marked shift from the usually observed pattern of socio-economic determinants of child mortality, in a population with high HIV prevalence and declining background levels of child mortality. In Karonga District in the 1990s we observed lower under-5 mortality in those whose fathers were traders or salaried workers compared to subsistence farmers or casual labourers, and lower mortality in those whose mothers had higher levels of education [13]. In the current study, factors such as higher maternal education, improved housing, and source of income, were not strong predictors of lower child mortality when mortality from all causes was considered.

Under-5 mortality in this population was 85 per 1000 births, lower than the national average estimate from the 2004 DHS (133 per 1000),[14] but comparable with levels recorded in 2004 in neighbouring Tanzania (83 per 1000 births).[15] Adult HIV prevalence has been around 12% since 2000 in Malawi [7] and in 2005 only 7% of HIV-infected mothers were estimated to have received ARV prophylaxis for the prevention of mother-to-child transmission.[7] Although the HIV epidemic is likely to have a severe impact on child mortality in Malawi, overall levels of mortality have steadily declined.[14] This can only be explained by a large decline in non-HIV related mortality, and with declining levels of background mortality, the relative impact of HIV is likely to increase.[16]

The comparative analysis of risk factors for AIDS- and non-AIDS mortality revealed that the absent or weak associations of socio-economic factors with child mortality from all causes were explained by the differential distribution of AIDS and non-AIDS deaths in the population. Almost all AIDS deaths in children in this population are likely to have been caused by transmission of HIV from mother to child and the distribution of paediatric AIDS deaths is therefore likely to mirror the distribution of HIV infection in women of reproductive age. It is possible that women of higher socio-economic status are more likely to be HIV tested, and knowledge of the mother's HIV status may have influenced classification of the death, but only about one third of the AIDS deaths in those under 5 years were in those whose mothers were known to be HIV-infected.

In contrast to the 2004 Malawi DHS, in which children of mothers with secondary school education had approximately 40% lower infant mortality and 60% lower under 5 mortality,[14] maternal education in the study population was only weakly associated with all cause mortality in infants or children. Infants and children of mothers with secondary education had the highest rates of AIDS mortality and the lowest rates of non-AIDS mortality, but both trends were weak. Trends with paternal education were also weak, and differed in infants and children. In both age groups there were high death rates in those with unknown maternal education, reflecting high mortality among maternal orphans (not shown).

Measurement of economic status at the household level in this study included ownership of household assets and dwelling construction, adapted to the local setting. Such indicators have shown close association with school enrolment and moderate correspondence with the traditional wealth indicators of household expenditure in India, Indonesia, Pakistan and Nepal,[17] and with HIV prevalence in sub-Saharan Africa.[18] In the current study, among 1–4 year olds, while there was no association of overall mortality with dwelling characteristics, there was a trend towards lower mortality with increasing asset value (*p* = 0.03). Separation into AIDS and non-AIDS mortality showed a clearer pattern, with high AIDS mortality in those with higher scoring dwellings, and high non-AIDS mortality in those with lower scoring dwellings and few assets.

For 1–4 year olds the pattern of deaths by household income source reflects the distribution of HIV in the population, with AIDS deaths dominating in households with employment, and non-AIDS deaths among those relying on subsistence farming. Higher HIV prevalence in wealthier adults (using quintiles derived from an asset-based wealth index) has been shown in DHS+ surveys from 8 countries in sub Saharan Africa, including in Malawi. [18]

There are no clear patterns for association with the household level measures for infants either overall or when divided into AIDS and non-AIDS mortality. This may reflect the greater difficulty of accurate assignment of cause of death as AIDS or non-AIDS in this age group. The sensitivity and specificity of verbal autopsy for the classification of infant and child AIDS deaths are poor,[19] [20] [21] although better when, as here, the autopsy interview is carried out soon after death. [22] The resulting misclassification is likely to have weakened the estimated effects of factors that had differential associations with AIDS and non-AIDS mortality. It is also possible that better-off HIV positive children are more likely to survive beyond infancy, reducing the association between socio-economic status and AIDS deaths in the infant age group, and magnifying it in the 1–4 year olds from the deferred deaths.

There was evidence that registration of births and deaths was near complete, from the re-census, from scheduled follow-ups of births after 1 year and from the distribution of ages at death. This makes it unlikely that child deaths were selectively missed (e.g. from poorer households and in remote areas). Parental and household characteristics were obtained by linking to other individuals in the study population. 95% of the mothers and 85% of the fathers of children below 5 years of age whose parents were not known to have died at baseline were among the population under surveillance, resulting in a high degree of completeness of information on parents. Asset score and main source of income were determined by interview, while dwelling construction was determined by direct observation by the field staff.

It is possible that other factors led to the lack of association between socio-economic status and child mortality. Differential migration could affect the trend if, for example, better off individuals stay in the district because of sickly children, but migration could affect all socio-economic levels.

The finding of the expected trends of higher non-AIDS mortality in children of households with few assets, poor dwelling construction, and relying on subsistence farming, suggests that the socio-economic indicators are measuring what they are supposed to measure. The lack of association with overall mortality is explained by the different trends in AIDS deaths. AIDS mortality was much higher in children from higher socio-economic status households, mirroring the distribution of HIV infection in the adult population. The usual and unwelcome gap in survival between the poor and the less poor has been lost, but because the less poor have been disproportionately affected by HIV, rather than because of relative improvement in the survival of the poorest.

Since HIV infection is associated with relative wealth in many parts of sub-Saharan Africa, [18] a change in the distribution of child mortality is likely in other settings. The extent to which it is apparent will depend on the relative contribution of AIDS and non-AIDS deaths, so it is most likely in settings and age groups in which background mortality rates are low.

It is probably a temporary phenomenon. A shift in the distribution of HIV in the population towards the poorer individuals has been predicted, [18] and a shift from the most educated groups earlier in the epidemic, to the less educated later, has been seen in several sub-Saharan African populations.[6] The survival gap is likely to return, and uneven access to antiretrovirals is likely to exacerbate the divide.
